# Ion Mobility Mass Spectrometry for Large Synthetic Molecules: Expanding the Analytical Toolbox

**DOI:** 10.1021/jacs.4c00354

**Published:** 2024-03-18

**Authors:** Niklas Geue, Richard E. P. Winpenny, Perdita E. Barran

**Affiliations:** † Michael Barber Centre for Collaborative Mass Spectrometry, Manchester Institute of Biotechnology, Department of Chemistry, The University of Manchester, 131 Princess Street, Manchester M1 7DN, U.K.; ‡ Department of Chemistry, The University of Manchester, Oxford Road, Manchester M13 9PL, U.K.

## Abstract

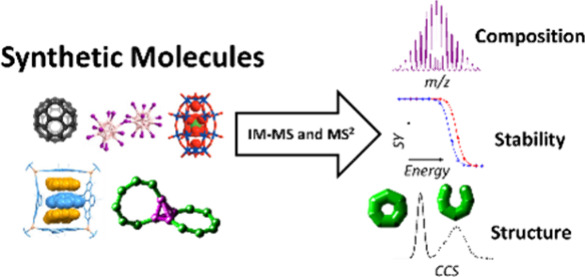

Understanding the composition, structure and stability of larger synthetic molecules is crucial for their design, yet currently the analytical tools commonly used do not always provide this information. In this perspective, we show how ion mobility mass spectrometry (IM-MS), in combination with tandem mass spectrometry, complementary techniques and computational methods, can be used to structurally characterize synthetic molecules, make and predict new complexes, monitor disassembly processes and determine stability. Using IM-MS, we present an experimental and computational framework for the analysis and design of complex molecular architectures such as (metallo)supramolecular cages, nanoclusters, interlocked molecules, rotaxanes, dendrimers, polymers and host–guest complexes.

## 1 Introduction

The successful synthesis of any molecule requires precise, robust and inexpensive sample characterization.^1^ Three different techniques are commonly used, namely X-ray crystallography, NMR spectroscopy and mass spectrometry (MS). These methods each can have drawbacks: the increasing difficulty of characterizing larger complexes, e.g. often due to poor crystallization (X-ray) or complex, overlapping signals (NMR), challenges with common methods of ionization, in source fragmentation and ion transmission (MS including ion mobility) and the inability to employ these techniques routinely with confidence on compounds produced at an industrial scale.^2^ In the discovery of new synthetic compounds, there is also an increased deployment of *ab initio* computational methods, but far less use of data from analytical methods that could be used to predict the analyte of interest based on particular physiochemical properties.^3,4^ While showing some promise, computational approaches to this problem are nontrivial and the required methodologies for large architectures are lacking.

Modern mass spectrometry (MS) is well suited to overcome the experimental challenges in analyzing large synthetic complexes,^2^ and can provide large, robust and reproducible data sets with high throughput capabilities, which will in turn inform predictive computational design.^5,6^ As ions are separated by their mass to charge ratio, which is determined by their chemical composition, the mass of a given analyte can be predicted and compared to measurement. Due to the high accuracy and resolving power of modern mass analysers, overlapping signals rarely occur, except for isomeric species with the exact same chemical composition, and the possibility of high-throughput (as applied in “omics” workflows) makes mass spectrometry an applicable tool to study synthetic products in industry.^171^

Over the past two decades, the commercialization of certain techniques has further improved the standing of mass spectrometry among its analytical competitors: (1) implementation of better ion optics for soft ionization techniques such as (nano)-electrospray ionization (ESI and nESI), cryospray-ionization (CSI) and matrix-assisted-laser desorption ionization (MALDI), facilitating the transfer of low concentration labile analytes and intermediates to the gas phase;^7^ (2) hyphenation with gas and liquid chromatography for additional separation; (3) developments in tandem mass spectrometry (MS^2^), which better enable the characterization of products from activated ions;^8^ and (4) the addition of ion mobility (IM) to mass spectrometers, enabling the separation of ions not only based on their *m*/*z*, but also on their charge, shape and size (Table 1).^9,10^ The focus of this perspective will be on the use of ion mobility coupled to mass spectrometry (IM-MS).

**Table 1:**
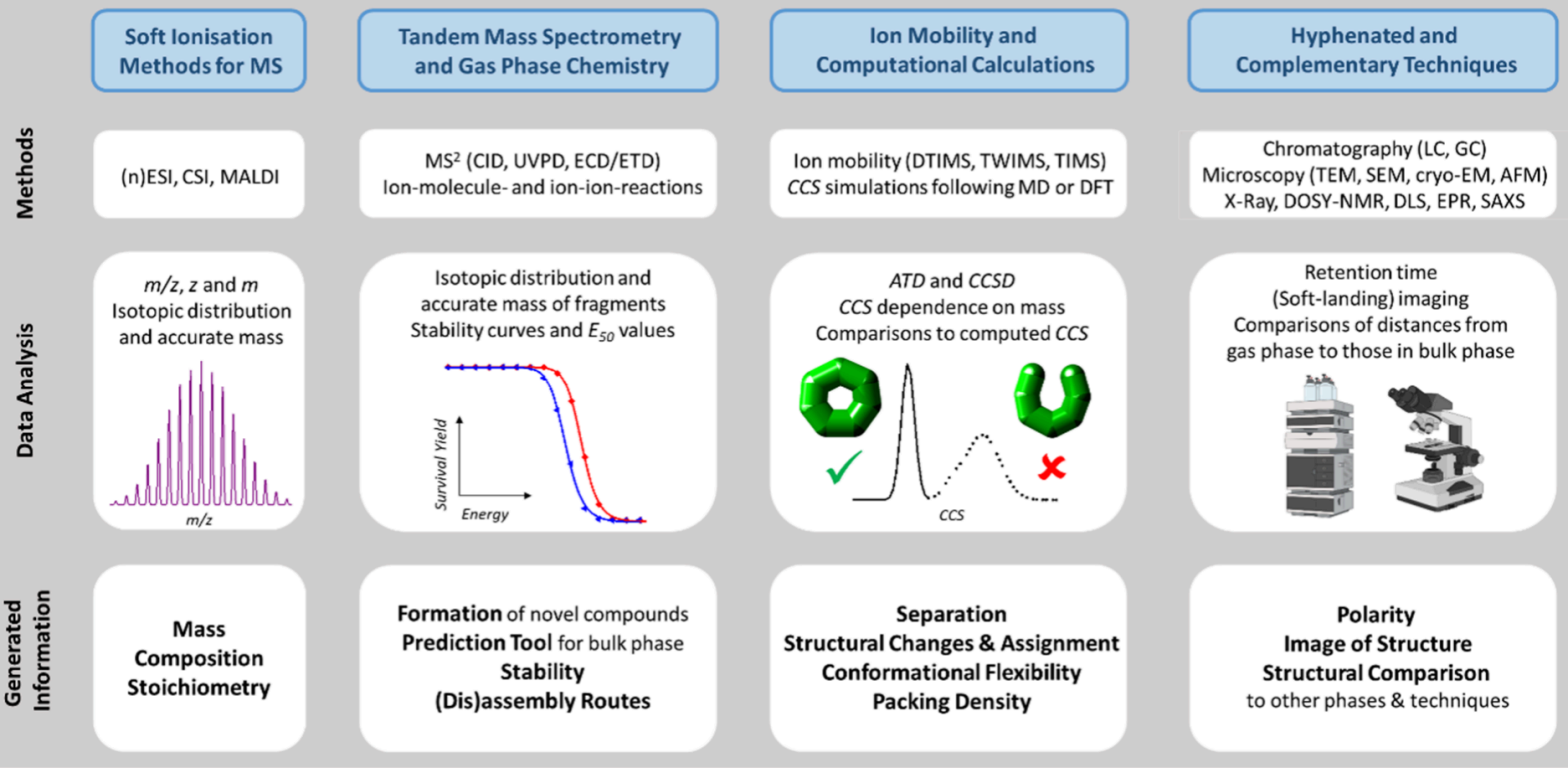
Advanced Mass Spectrometry Methods Appropriate for Large Synthetic Molecules^a^

a Characteristic methods, their data analysis and the generated information are displayed. Partially designed with BioRender. Reproduced with Permission under a CC-BY 4.0 License from ref (57), © 2023 Springer Nature.

In two of the most common IM-MS methods, drift tube IM and traveling wave IM, ions traverse a gas-filled cell under the influence of weak electric fields, wherein they undergo many collisions with the gas.^10^ For ions of the same charge, larger and more elongated species need more time to pass through the mobility cell as they experience more ion–gas collisions. An alternative method is trapped IM, which operates in the opposite way. Ions are pushed by a gas flow against an electric field, and are separated based on the field that is needed to resist the gas flow. In general, the time each ion spends in the cell is measured and is a so-called drift time (or arrival time at the detector), which can be converted to a rotationally averaged collision cross section (*CCS*).^10^

The *CCS* of any given ion is a composite parameter that describes the size, shape and charge of the ion as well as how it interacts with the gas. It is commonly reported as *CCS*_*He*_ or *CCS*_*N2*_, referring to a measurement made in helium or nitrogen as buffer gas, respectively. The *CCS* of any given ion provides structural information and can be compared between instruments and laboratories, as well as to *CCS* values predicted computationally from candidate structures.^9,11^ With the introduction of the first commercial IM platform in 2006, the use of IM-MS has expanded rapidly to research and industry laboratories worldwide with applications in separation of complex mixtures and structure determination.^12,13^ The latter has primarily been beneficial for biological systems, including proteins and protein complexes, nucleotides, antibodies, lipids, carbohydrates and viruses.^10^ For other, nonbiological systems, IM-MS has been underexplored, despite the potential demonstrated for structural biology.

The use of mass spectrometry methods for supramolecular complexes^2,14−19^ and polymers^14,20−22^ has been regularly reviewed, however usually from the perspective of mass spectrometrists with a particular interest in these systems. Here, we focus on recent developments and their implications for synthetic chemists, highlighting the opportunities of IM as an analytical tool of the future. We explore how synthetic complexes and architectures can be generated using *in vacuo* methods, particularly if they are not easily amenable by other means, and we emphasize how these discoveries translate to chemistry in the bulk phase. The second part focuses on how synthesized molecules can be structurally characterized with IM-MS, e.g. via computational modeling, and how IM-MS data can be used to draw conclusions on the analytes’ general topology, cavity size, conformational dynamics and packing density. We highlight the latter and compare density across compound families, illustrating how molecular architecture determines physicochemical ion properties. In the last section, we discuss the application of IM-MS for analyzing the stability and disassembly pathways of gas phase ions, and how these data can inform the tools used by synthetic chemists.

## 2 Discovery and Formation of Novel Synthetic Molecules in *Vacuo*

The use of mass spectrometry, particularly in combination with (n)ESI, has led to the discovery of compounds in the gas-phase that were at the time, or even still, not readily observable using solution-phase techniques. This can have different explanations: the complexes form in solution, but are intermediates, too labile or not abundant enough to be measured with bulk-phase techniques or harsher ionization MS techniques, or these architectures are formed directly in the gas phase via adduction (e.g., oligomerization) or by reactions with other gas phase ions/molecules.

### 2.1 Gas Phase Chemistry as an Informative Tool for Bulk Phase

**Figure 1:**
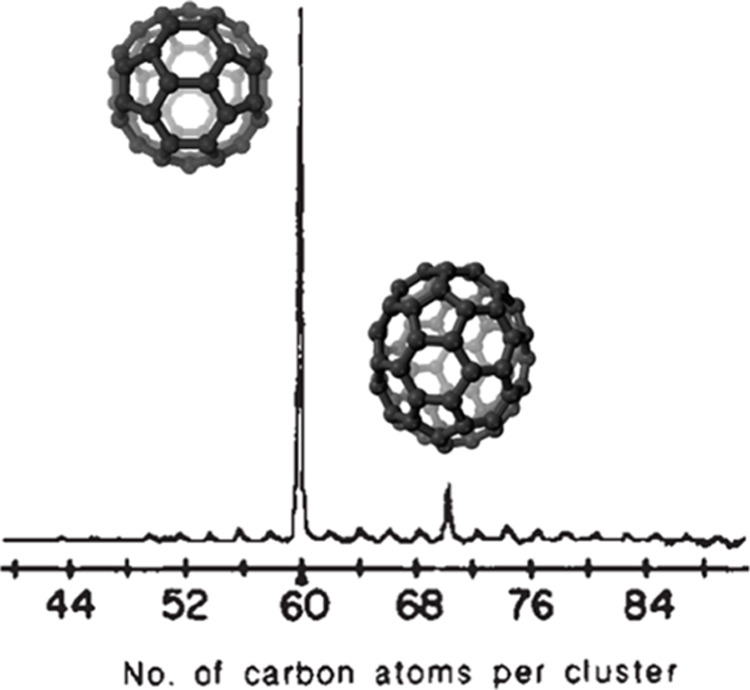
Mass spectrum of carbon clusters prepared by laser vaporization of graphite and cooled in a supersonic beam including schematic structures of the C_60_ and C_70_ fullerenes. Reproduced from ref (26) with permission from Springer Nature, © 1985.

Perhaps the most famous example of a molecular assembly shown to have high stability in the gas phase, and later synthesized in the bulk phase, is Buckminsterfullerene C_60_. In their work from 1985, which was later awarded the Noble Prize in Chemistry,^23−25^ Kroto et al. vaporized different carbon species from the surface of a graphite disk, ionized the carbon clusters with an excimer laser, and subsequently mass-analyzed the ions.^26^ The spectra showed higher intensities for the C_*n*_ clusters with *n* = 60, and less pronounced *n* = 70, suggesting that these species are more stable than others (Figure 1). Five years later, Krätschmer et al. confirmed these experiments by synthesizing C_60_ for the first time in the bulk phase, and here minor C_70_ levels were present as well.^27^ Shortly after, Bowers, Jarrold, von Helden, and co-workers used IM to investigate the formation mechanism of fullerenes in the gas phase, showing that collisional heating (as discussed further below) allows carbon rings to react and form fullerenes.^28,29^

Cronin and co-workers made progress in utilizing cryospray-ionization mass spectrometry (CSI-MS), a particularly soft ionization method operating at lower temperatures, as well as ESI-MS to follow the self-assembly of polyoxometalates (POMs).^30−33^ POMs are anionic, nanosized particles with many aspirational applications, e.g. in molecular electronics, catalysis, energy storage and medicine,^34−36^ and one of their most promising properties is that they are transferable building blocks. Understanding and controlling their assembly is hence highly desirable for the targeted synthesis of more complex POM architectures.^37^ Along the way of following their self-assembly routes, the Cronin group used CSI- and ESI-MS to scan solutions for potentially novel cluster types, and this workflow led to the discovery of the novel cluster types {M_17_V_3_} (M = Mo, W),^38^ {Mo_11_V_7_},^39^ {W_18_Te},^40^ and {Ag_2_Mo_8_}_*n*_,^30^ among others.

Reactions between gas phase ions and neutral molecules, and similarly between ions and ions as pioneered by McLuckey and co-workers,^41−43^ are well-known to produce novel species in the gas phase. For example, O’Hair, Rijs, Schwarz, Stace and others have applied this widely for organometallic species to investigate reactive intermediates with relevance for catalysis.^44−55^ A related approach to make new complexes in the gas phase is by collisionally fragmenting larger precursor ions using tandem mass spectrometry. We have previously applied MS^2^, more precisely collision-induced dissociation (CID), to produce polymetallic rings in the gas phase whose precise stoichiometry has to date been elusive for bulk-phase synthesis. In a CID experiment, ions are accelerated at user-defined kinetic energies into a cell filled with a collision gas, which leads to activation and often dissociation.

**Figure 2:**
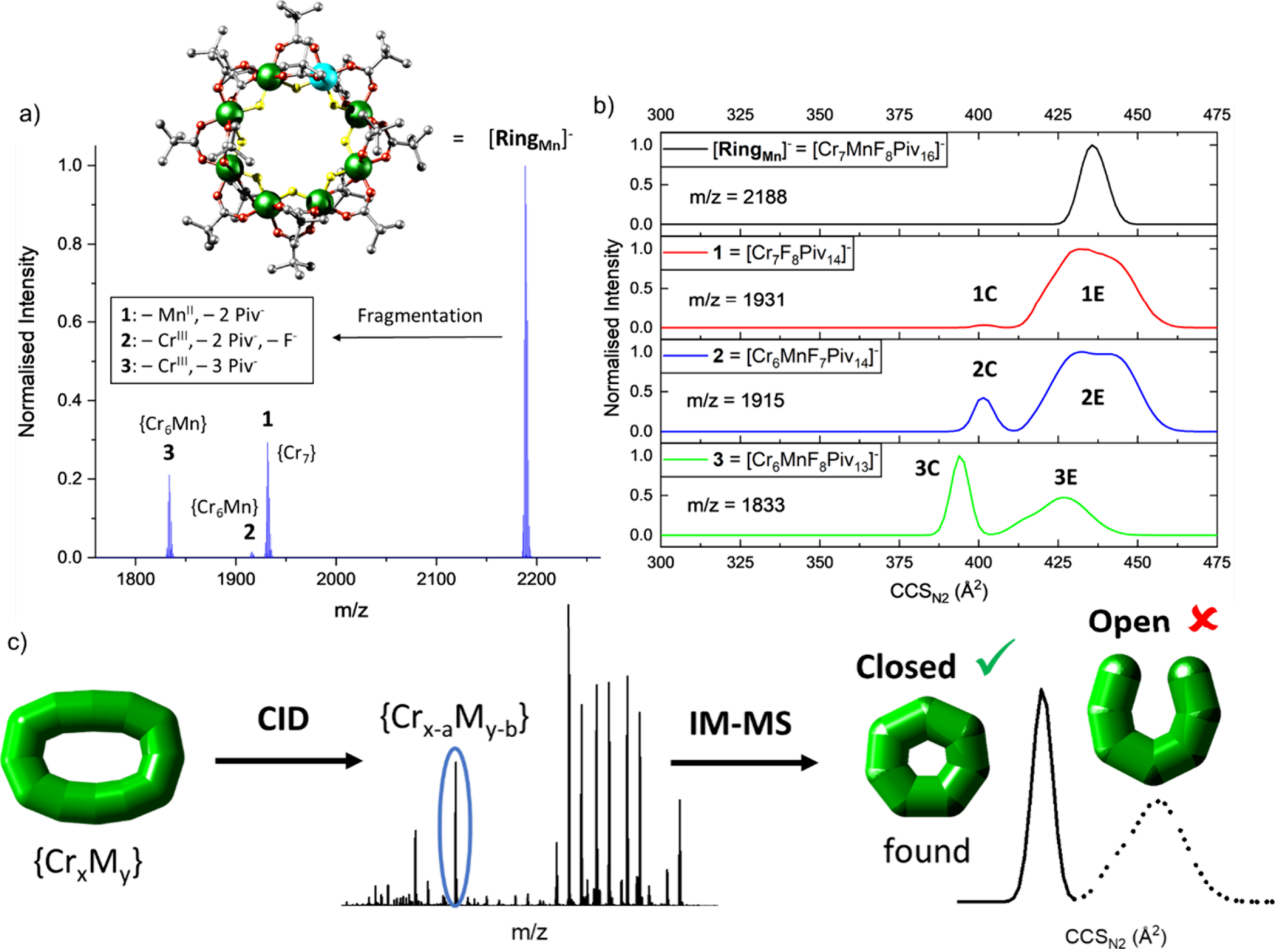
(a) CID spectrum of [**Ring**_**Mn**_]^−^, Inset: structure of [**Ring**_**Mn**_]^−^ (Cr: green, Mn: cyan, F: yellow, O: red, C: gray). Hydrogen atoms in the *tert*-butyl groups were omitted for clarity. (b) *CCS*_*N2*_ Distributions of [**Ring**_**Mn**_]^−^ and fragments (**1**–**3**). c) CID-IM-MS workflow following nanoelectrospray ionization of the precursor {Cr_*x*_M_*y*_}, more precisely {Cr_10_Cu_2_}, {Cr_12_Cu_2_} and {Cr_12_Gd_4_}, from solution and including *m*/*z*-selection of appropriate precursor ions. Upon collisional activation, several fragment ions are observed with different masses, and their CCS_N2_ distribution can be extracted as shown for an ion of the type {Cr_x-a_M_y-b_}. Different structures of {Cr_x-a_M_y-b_} are possible in theory, e.g. closed (low *CCS*_*N2*_, narrow *CCS*_*N2*_ distribution) or open forms (high *CCS*_*N2*_, wide *CCS*_*N2*_ distribution), of which only a closed species was found in this hypothetical example (continuous line). Reproduced from ref (56), © 2022 The Authors, and ref (57), © 2023 Springer Nature, with permission under CC-BY 4.0 licenses.

We have applied CID to {Cr_7_M} ring anions ([Cr_7_MF_8_(O_2_C^*t*^Bu)_16_]^−^ = [Ring_M_]^−^, with M = Mn^II^, Fe^II^, Co^II^, Ni^II^, Cu^II^, Zn^II^, and Cd^II^), in which the metal centers are bridged with fluoride and pivalate ligands (O_2_C^*t*^Bu^–^ = Piv^–^).^56^ This led to the loss of metal centers as the first fragmentation step, yielding both {Cr_7_} (**1**) and {Cr_6_M} (**2**, **3**) product stoichometries (Figure 2a). IM was used to identify these as a mixture of closed complexes and open, conformational dynamic “horseshoe” structures (Figure 2b for M = Mn^II^, see discussion below).^56^ As heptametallic rings are interesting synthetic targets, and hard to make in solution, we aimed to shift the closed:open topology ratio further toward the former. This was achieved when collisionally activating larger, but similar compounds of the types {Cr_*x*_Cu_2_} (*x* = 10, 12) and {Cr_12_Gd_4_}, in which the metal centers are analogously bridged via Piv^–^ and F^–^ ligands; and this workflow produced almost exclusively rings (Figure 2c).^57^ Among others, the heptametallic species {Cr_6_Cu}, {Cr_5_Cu_2_} and {Cr_5_Gd_2_} were formed, as well as a range of larger rings.

As disassembly led to the formation of smaller polymetallic rings from all three precursors, we suggest that this may be a general phenomenon and that polymetallic rings with varying sizes and stoichiometries, including new types, are stable and can be made *in vacuo* as long as larger precursors are available. The MS^2^ spectra exhibited also some notable gaps, and similarly to that shown for fullerenes by Kroto et al. (Figure 1), and supported by studies from Barran et al.^58^ as well as Laskin and Lifshitz,^59^ this implied stability inspired us to target and improve the solution synthesis for some of the gas phase formed complexes.

### 2.2 Connecting Gas Phase with Bulk Phase: Ion Soft-Landing

A common question that mass spectrometrists face frequently is how relevant such gas phase studies are to bulk phase chemistry? One advantage is what we can learn about the ions’ stability relative to other species, and this knowledge can potentially be applied to studies in solution. It might also not be necessary to investigate molecules in bulk, as only a minority of compounds will ever be produced on a large scale. For example, ions of interest could be further investigated in the gas phase, e.g. via MS^3^ platforms^60^ where product ions can be isolated again and activated further,^61−64^ or examined spectroscopically.^17,60,65^ Such approaches, in combination with IM, can potentially inform on their structure, disassembly and stability, without producing them in bulk. However, stability trends do not necessarily correlate between gas and condensed phases, and some ions could not even be stable at all outside the mass spectrometer.

**Figure 3:**
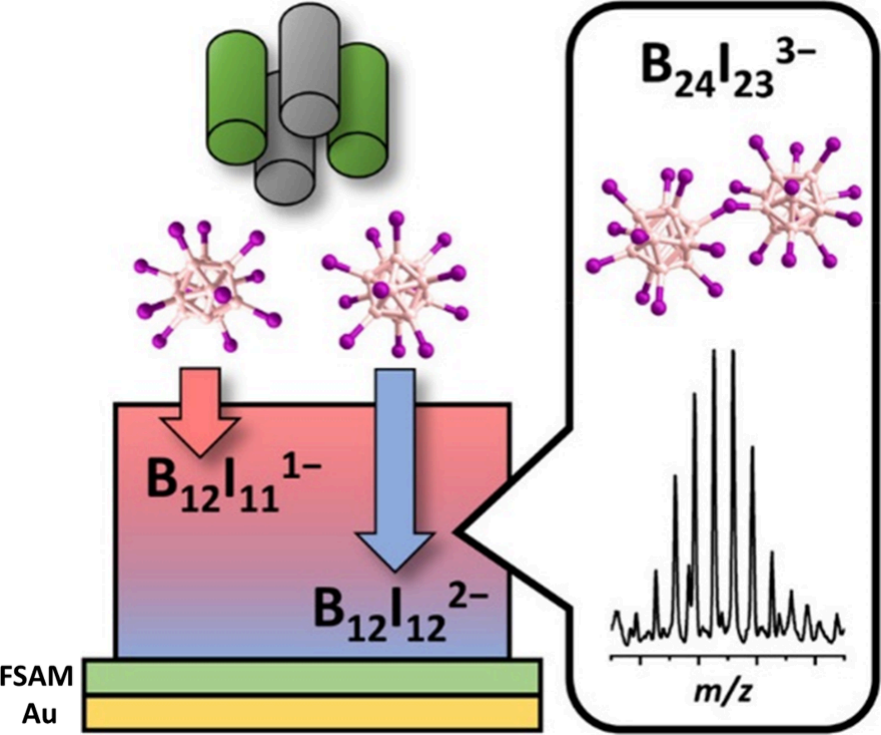
Schematic of the anion–anion reaction between [B_12_I_12_]^2–^ and [B_12_I_11_]^−^ on a gold surface with a fluorinated alkane-thiol self-assembled monolayer (FSAM). This led to the formation of [B_24_I_23_]^3–^ as confirmed by mass spectrometry. Reproduced from ref (70) with permission, © 2021 The Authors.

Another way of connecting gas phase with solution is preparative mass spectrometry (“ion soft-landing”), where gaseous ions are deposited on a solid surface and characterized, often with microscopy.^66−69^ It is also possible to use ion soft-landing to examine reactions at surfaces: Yang et al. used preparative mass spectrometry explored reactions between ions of the same polarity, namely the reactive [B_12_I_11_]^−^ with the unreactive [B_12_I_12_]^2–^ (Figure 3).^70^ The latter was deposited on a modified gold surface, before the former was soft-landed on the [B_12_I_12_]^2–^ enriched layer. This led to the formation of the product [B_24_I_23_]^3–^, showing that highly charged clusters can be formed directly from gaseous building blocks. To date, the ions per time deposition numbers achieved are too low for this workflow to be an actual alternative for synthetic methods in solution; however, the road ahead has promise.^172^ An important milestone would be the coupling with IM, enabling a better synthetic control by depositing both *m*/*z*- and conformationally selected ions.^10^

## 3 Structural Characterization Using Ion Mobility

The main goal of using IM-MS for synthetic molecules is to characterize their structure, and this is pertinent for systems where other analytical techniques such as X-ray crystallography or NMR spectroscopy are not feasible or do not provide sufficient information (e.g., often for large supramolecular complexes).^2^ One of the main advantages of IM-MS is that many target analytes can be investigated simultaneously even from complex mixtures, whereas common bulk phase techniques often only provide the averaged response of all species present. This is of particular interest when screening the products of self-assembly reactions, where multiple stoichiometries, structures and conformations are present. In their pioneering works, Wesdemiotis, Newkome and co-workers demonstrated this for metallosupramolecular complexes,^71−74^ and since then many groups with similar synthetic targets, such as Chan,^75−78^ Clever,^79−83^ Li,^84−90^ and Nitschke,^91−94^ have deployed IM-MS as a standard analytical technique.

### 3.1 Separating Isomers, Stereoisomers and Electronic States

One of the main advantages of IM is the ability to resolve highly similar ions by measuring differences in arrival time or *CCS*, and this often depends on the resolving power of the instrument. The latter has increased over the past two decades, for example in the form of cyclical IM-MS^95,96^ and SLIM (structures of lossless ion manipulation) mobility devices,^97^ and we expect that such developments in instrumentation will further drive our ability to separate ions based on their mobility.^98^ As early as 1990, using a drift-tube IM-MS instrument, Kemper and Bowers were able to separate the ground and excited state of Co^+^.^99^ Recently, Mullen and co-workers separated the diastereomers of different {M_4_L_6_} coordination cages.^100^

The separation of isomers and even enantiomers^101^ is a particular interesting opportunity of IM-MS. While enantiomers cannot be separated directly using conventional IM, this can for example be accomplished via the formation of host–guest complexes with a chiral macrocyclic selector. Here the macrocycle can work as an IM shift reagent, and differences in noncovalent complexation facilitate or even enable the separation with IM.^19^ Isomers of bile acid,^102^ enantiomers of ibuprofen and flurbiprofen,^103^ and enantiomers of 19 amino acids^104^ have been separated upon encapsulation in cyclodextrin macrocycles, and other IM shift reagents have also been used.^19^ Since the development and manufacturing of new pharmaceutical entities relies on producing pure isomers and enantiomers, IM-MS in combination with such shift reagents has potential for applications in the screening and separation of drug candidates.

### 3.2 Distinguishing Different Binding Sites

**Figure 4:**
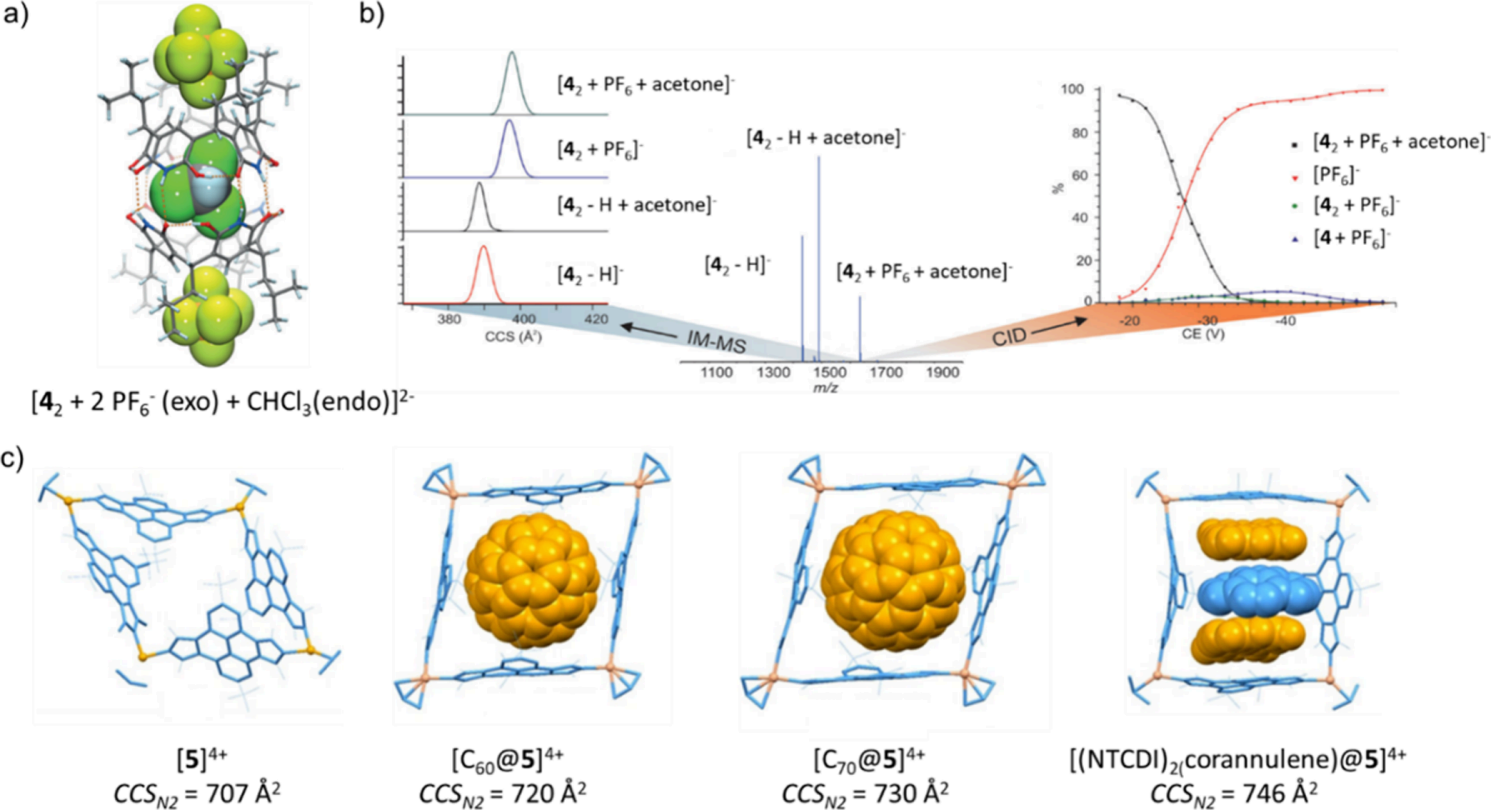
(a) Crystal structure of [**4**_2_ + 2 PF_6_^–^_(exo)_ + CHCl_3(endo)_]^2–^. PF_6_^–^ and CHCl_3_ are shown in spacefilling mode and the dashed orange lines represent hydrogen bonds. (b) IM spectra for selected ions of **4**_**2**_, center: ESI-MS spectrum of **4** with an equimolar amount of NH_4_PF_6_ in acetone, right: CID curves for the ternary complex [**4**_2_ + PF_6_ + acetone]^−^. (c) DFT optimized structures of [**5**]^4+^, [C_60_@**5**]^4+^ [C_70_@**5**]^4+^ and [(NTCDI)_2_(corannulene)@**5**]^4+^ and their *CCS*_*N2*_ values. Reproduced from ref (105) with permission, © 2017 Wiley-VCH Verlag GmbH & Co. KGaA, and from ref (109) under a CC-BY 4.0 license, © 2021 The Authors.

IM-MS can also be applied to investigate binding site interactions. Kiesilä et al. examined the complex formed between a pyridine[4]arene dimer, hexafluorophosphate anions and organic solvent molecules (Figure 4a, b).^105^ Two binding sites are possible in the dimeric host **4**_**2**_, *exo* and *endo*. The IM experiment yielded a highly similar *CCS*_*N2*_ value for the binary complex [**4**_2_ + acetone]^−^ and the isolated dimer [**4**_2_ – H]^−^, whereas a significant *CCS*_*N2*_ increase is observed for the complexes [**4**_2_ + PF_6_]^−^ and [**4**_2_ + PF_6_ + acetone]^−^. This suggests that PF_6_^–^ is *exo* coordinated and acetone *endo*. More evidence came from MS^2^ of the ternary complex [**4**_2_ + PF_6_ + acetone]^−^, which showed the loss of PF_6_^–^ in the initial fragmentation step, suggesting *exo* complexation of PF_6_^–^. The higher binding strength of the neutral acetone to **4**_**2**_ suggests in turn *endo* complexation.

### 3.3 IM-MS as a Tool to Measure Conformational Flexibility and Structural Dynamics

Besides the absolute *CCS* value, the width and shape of a given *CCS* distribution can often inform on the conformational flexibility of the system. Asymmetry in the peak shape can indicate unresolved conformers or conformational flexibility. If the peaks are symmetric or only slightly asymmetric, and no further conformers can be resolved even with high resolution IM, the full width at half-maximum (FWHM) is a useful tool to assess conformational flexibility. In our previous work, we found that the FWHM for a series of rotaxanes involving [**Ring**_**M**_]^−^ (M = Mn^II^, Fe^II^, Co^II^, Ni^II^, Cu^II^, Zn^II^, Cd^II^) depends on M, with smaller values for Mn^II^ and Cd^II^.^106^ This is likely correlated to their larger radii, leading to a more rigid structure as discussed in detail in our work.^106^ Care should be taken when quantifying peak widths of IM data, as for example space-charge artifacts can influence the peak widths.^107^

The application of IM-MS to examine conformational change is obtained by measuring differences in arrival time or *CCS* between related species. For example, Nau and co-workers used IM-MS in combination with MS^2^ and DFT to understand the gas-phase chemistry of azoalkanes encapsulated in cucurbiturils.^108^ The authors found that the reactivity of the guest within the host strongly depends on the volume of activation for the reaction of interest as well as the void space in the macrocycle, and these findings are likely transferable to catalysis and reaction dynamics in confined reaction spaces.

In a more recent study, Peris and co-workers tracked distortions of hosts and guests in organometallic metallosquares using IM-MS (Figure 4c).^109^ A tetracationic palladium square **5**, involving four pyrene-bis-imidazolylidene ligands, was used to encapsulate C_60_ and C_70_ fullerenes as well as naphthalenetetracarboxylic diimide (NTCDI) and a series of polycyclic aromatic hydrocarbons (PAHs). IM showed that the organometallic square **5** (*CCS*_*N2*_ = 707 Å^2^) significantly expanded when encapsulating C_60_ (*CCS*_*N2*_ = 720 Å^2^) and C_70_ (*CCS*_*N2*_ = 730 Å^2^), which was in agreement with results from density functional theory (DFT). **5** was also used to form host–guest complexes with three stacked heteroguests of the formula [(NTCDI)_2_(PAH)@**5**]^4+^, which exhibited slightly larger, but constant *CCS*_*N2*_ values with varying PAHs (*CCS*_*N2*_ = 744–746 Å^2^). This indicated similar dimensions of the different guests, which was noteworthy for the corannulene that is bowl-shaped in contrast to the other PAHs. The authors suggested that the formation of the host–guest complex in combination with the metallosquare **5** results in the flattening of corannulene, which was again supported by DFT calculations (bowl-depth: 0.90 Å (isolated); 0.78 Å (host–guest complex)).

Structural dynamics are of high interest to synthetic chemists, particularly for molecular machines and macrocycles, where motions caused by external stimuli (e.g., light, solvent, temperature) are exploited.^110^ Schalley and co-workers used IM-MS, among other techniques, to show the light-induced interconversion of a crown-ether complex between a [c2]daisy chain and a lasso-type pseudo[1]rotaxane.^111^ Urner et al. tracked the kinetics for the *cis*/*trans* isomerization of an azobenzene-based dendritic bolaamphiphile by irradiating the sample in the nanoESI source, illustrating that IM-MS can be used to follow reaction dynamics live.^112^ Warzok et al. demonstrated a structural rearrangement from a hexameric to a pentameric halogen-bonded capsule using IM-MS, when changing the solvent from chloroform to dichloromethane.^113^

**Figure 5:**
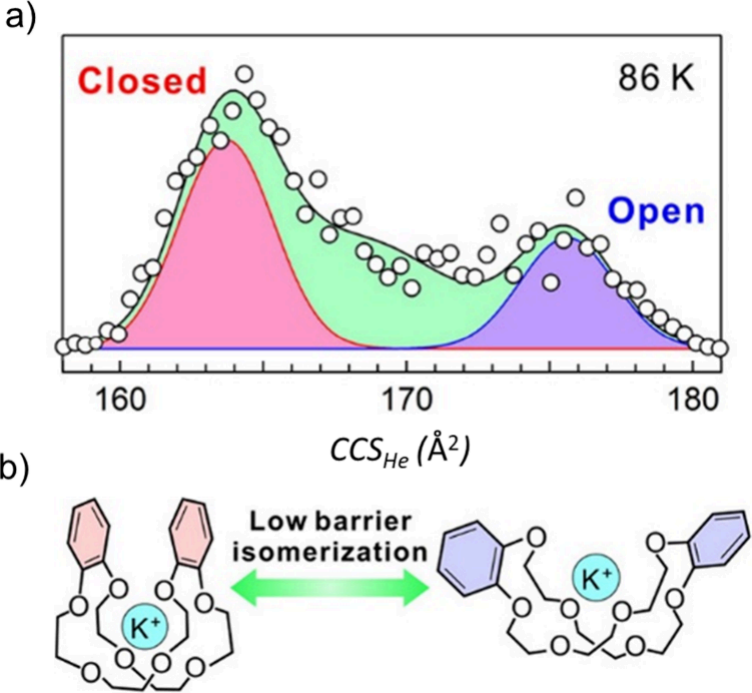
(a) *CCS*_*He*_ distribution of [K@DB24C8]^+^ at 86 K, which show the presence of a closed (red) and an open (blue) conformation after Gaussian fitting. Experimental data points are shown as black circles, and the total distribution is fitted (green). (b) Schematic of the isomerization reaction between the closed and open form of [K@DB24C8]^+^_._ Reproduced from ref (121), © 2022 American Chemical Society.

Temperature can also alter conformation, and this can be directly investigated with variable temperature IM-MS (VT IM-MS), primarily demonstrated with biomacromolecules.^114−120^ Ito et al. recently studied the conformational changes of dibenzo-24-crown-8 (DB24C8) with four guest cations (Ag^+^, Na^+^, K^+^, NH_4_^+^) using VT IM-MS, showing that both a closed and open conformation can be resolved at 86 K (Figure 5 for K^+^), as opposed to higher temperatures.^121^ In combination with DFT calculations, the isomerization rate constant was determined at several temperatures, and the activation energy for the interconversion reaction between both populations was found to be relatively low (*E*_*A*_ = 4.8–9.0 kJ/mol^–1^). Reaction pathway calculations additionally revealed the detailed isomerization mechanism in combination with DFT and *CCS* calculations, highlighting the powerful interplay of IM-MS and computational resources.

### 3.4 Structural Assignments Guided by Computational Modeling Coupled to IM-MS

Modeling theoretical *CCS* values is a powerful approach to directly probe the conformational landscape observed experimentally with IM-MS, and for that purpose a range of methods and programs have been developed over the past decades.^122,123^ One of the more challenging aspects in these computational approaches is to simulate the interaction of gas with different molecular topologies, such as concave surfaces and cavities where multiple collisions may occur. The trajectory method (TM) has the closest representation of experiment and considers both short- and long-range interactions as well as multiple collision events, whereas other methods, such as the projection approximation or exact hard sphere scattering, are more approximate and rely on empirical inputs from a narrow range of chemical classes.^122−124^ The precision of theoretically derived *CCS* values, compared to experiment, is relatively high for small molecules; however, this does not always exclude other candidate structures that may have similar nominal *CCS*.^10^ Another problem is that the parametrization of elements beyond those commonly found (H, C, N, O, F, Na, Si, S, Cl, K) is not yet well explored and can lead to notable deviations in theoretical *CCS*, as well as not taking hollow architectures into account properly.^56^

Structures input for theoretical *CCS* calculation can be obtained in different ways, sorted by increasing accuracy and computational cost: built from scratch using a molecular editor,^125^ directly from X-ray crystallography,^126^ sampled with molecular dynamics (MD) simulations^127^ which often yield less compact structures than experiment,^128,129^ and/or geometry optimized with quantum chemical methods.^56^ The choice of the method depends on the analyte, available computational resources and the feasibility of the different methods; in general computational modeling can be laborious when high precision in comparison to experimental values is sought.

Our perspective on the future of *CCS* value prediction is the use of artificial intelligence methods and subsequent comparison with experiment. This has recently begun to be realized in metabolomics,^6,130,131^ and even for synthetic molecules the number of measured *CCS* values has rapidly increased in recent years, and is or will soon be sufficient to begin with the training of machine learning algorithms.

In a study by Cronin and co-workers investigating a series of nanosized POMs, the comparison between experimental *CCS* values to those modeled from crystal structures yielded significantly higher experimental values with a discrepancy of up to 28%.^126^ This was attributed to inappropriate Lennard–Jones parameters of the transition metals used, as well as to the lack of partial charges and counterion involvement. It should be noted that proteins systematically tend to show the opposite trend, where the predicted *CCS* value from crystal structure is often significantly larger than experiment (ca. 10–30%, depending on protein size).^122^ This is attributed to contraction of the structure upon removal of solvent molecules and counterions. We suggest that this is less likely to occur with large synthetic molecules, stable in aprotic solvents, although the magnitude of any contraction will depend on the specific structure and rigidity.^132^ Modeling *CCS* values directly from crystal structures without further refinements has major limitations, and is not recommended.^2^

**Figure 6:**
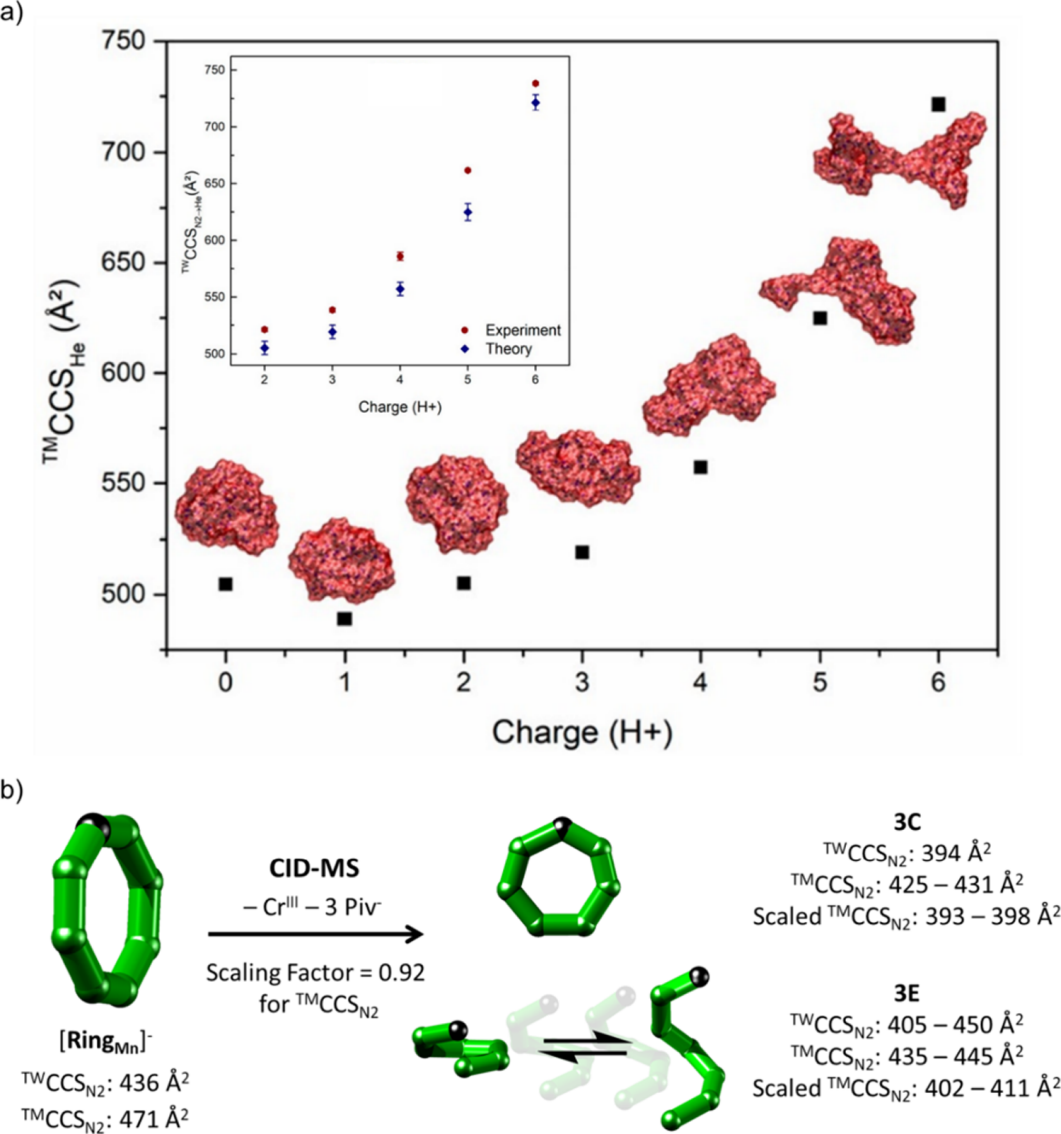
(a) Evolution of the *CCS*_*He*_ for the 2nd generation of a PAMAM dendrimer from 0 to 6 charges. The structures are represented with the van der Waals surface (red). Inset: Comparison between experimental (red circle) and theoretical (blue diamond) *CCS*_*He*_ for the 2nd generation of a PAMAM dendrimer. Error bars represent the standard deviation on three experimental measurements and on 200 theoretical structures, respectively. (b) Workflow for the assignment of DFT optimized structures to **3C** and **3E**. Comparison of the theoretical ^*TM*^*CCS*_*N2*_ and experimental ^*TW*^*CCS*_*N2*_ values of [**Ring**_**Mn**_]^−^ yielded a difference of 8%, and hence a scaling factor of 0.92 was applied for the assignment of **3C** and **3E**. Reproduced from ref (127) with permission, © 2020 American Society for Mass Spectrometry as well as from ref (56), © 2022 The Authors.

More advanced is the comparison to *CCS* values modeled from molecular dynamics (MD) simulations, as applied by Brocker et al. for a series of rigid triangular, rectangular and prismatic platinum coordination assemblies.^133^ Using the ESFF force field,^134^ parametrized to incorporate transition metals with varying coordination geometries, annealing was simulated for the ions studied, after updating the bond distances with those from X-ray structures. Subsequent *CCS*_*He*_ modeling, using both the simple projection approximation (PA) and TM, showed good agreement with experimental *CCS*_*He*_ values. In another study, Gerbaux and co-workers used MD simulations to assign the structures of poly(amidoamine) (PAMAM) and poly(propyleneimine) (PPI) dendrimer families.^127^ Dendrimers are hyperbranched polymers, similar to fractals, and occur in different generations, which are synthesized stepwise from the core to the edge of the structure. In solution, these architectures often adopt globular morphologies; however their structure in the gas phase strongly depends on the generation and charge state *z* of the corresponding ion. Applying MD simulations with the force field gaff2,^135^ the authors computed the structures of the second generation of a PAMAM dendrimer, and found good agreement between theoretical *CCS*_*He*_, based on the trajectory method, and experimental *CCS*_*N2→He*_ values for each charge state (Figure 6a). The combination of MD and TM revealed that increasing the charge state leads to a progressive elongation of the structure, which evolves from a sphere (*z* = 1) to an ellipsoid (*z* = 3). Comparison with the species for *z* = 5 and more pronounced for *z* = 6 shows that the dendrimer branches start to separate significantly at higher charge states, which goes along with a steeper increase in *CCS*. This qualitative trend was found for all dendrimer families and generations, suggesting that this evolution could apply to dendrimers in general.

Quantum chemical methods such as density functional theory (DFT) can also be used to model candidate structures for *CCS* comparison, and well-known is the work on small coin metal clusters of Kappes, Weis and others.^136−141^ Zimnicka et al. used DFT calculations to benchmark the relationship between experimental *CCS*_*He*_/*CCS*_*N2*_ and those computed with the trajectory method for a class of anionic and rigid tetralactam macrocycle adducts.^142^ The authors found high agreement for both gases (*ΔCCS*_*He*_ = 1.5% and *ΔCCS*_*N2*_ = 3.2%), demonstrating the suitability of DFT and the trajectory method for such architectures. In our recent work on the polymetallic rings [**Ring**_**M**_]^−^ (M = Mn^II^, Fe^II^, Co^II^, Ni^II^, Cu^II^, Zn^II^, Cd^II^),^56^ we used DFT to optimize structures for each {Cr_7_M} ring with the B3LYP level of theory and based on the crystal structure. The TM-modeled *CCS*_*N2*_ values of these were found to be 8% higher than experiment, and we suggested that the lack of transition metal parametrization as well as accounting improperly for the ring cavity are plausible explanations for this discrepancy. We further investigated the structure of the corresponding fragment **3** using the same DFT workflow applied to a series of hand-drawn candidate structures, including a closed {Cr_6_M} ring and several open helical conformers (Figure 6b). To account for the *CCS*_*N2*_ differences observed in [**Ring**_**M**_]^−^, we applied a scaling factor of 0.92 to the theoretical *CCS*_*N2*_ values. Experimentally, two *CCS*_*N2*_ distributions were found, one narrow at lower *CCS*_*N2*_ and one wider at higher *CCS*_*N2*_ (Figure 2b). The scaled *CCS*_*N2*_ value of the closed ring and a slightly opened conformer agreed well with the former, whereas the more open structures corresponded to the wider distribution. Hence, DFT can be used not only to characterize the structure of solution-synthesized complexes but also to probe the conformational landscape of novel fragments produced in the gas phase via MS^2^.

### 3.5 Comparison of IM-MS Data to Other Experimental Techniques

*CCS* values can be compared not only to computationally modeled data but also to size and distance measurements obtained from other experimental techniques. Based on the rough assumption of a given ion being spherical and homogeneous, neglecting other factors such as partial charge distributions and the type of drift gas, the *CCS* is correlated with the ion radius via the formula *CCS = πr*^2^. For the dimeric species **4**_**2**_ (Figure 4a), the radius derived from the *CCS* value was 2.2–2.3 nm, depending on counterions and solvent molecules, and comparison with the hydrodynamic radius from DOSY (*r* = 2.0 nm) or to the crystal structure radius (*r* = 1.9 nm) yielded acceptable agreement, although this comparison is not robust for small distance differences. The data at least suggests similar connectivities and conformations in all three phases, illustrating the applicability of IM-MS to understand bulk phase phenomena. Similar comparisons have been made with other analytical methods that can provide distance information, such as small-angle X-ray scattering,^143^ dynamic light scattering, double electron–electron resonance spectroscopy, or microscopy techniques.^2^ Combining IM-MS with other methods in the same experiment (hyphenation) is another promising route to maximize the information content on given analytes, and common examples include liquid and gas chromatography, gas phase spectroscopy, and even microscopy in the form of ion soft-landing (see the Connecting Gas Phase with Bulk Phase: Ion Soft-Landing section).

### 3.6 Relationship between *CCS* and Mass: Insights into Ion Packing Density

The correlation between the *CCS* and mass/number of subunits of a given series of ions can inform on structural trends. This is based on their packing density, which is largest for ions with high mass and low *CCS*.^144−146^ Bleiholder et al. tracked the self-assembly of peptides relevant for amyloid fibril formation and, based on their packing density, distinguished larger globular peptides from β-sheets.^145^ We also showed that the *CCS*/*m* slope of intrinsically disordered and unfolded proteins is higher (lower packing density) than for proteins in their native state.^146^ More recently, McLean and co-workers built a database based on this correlation, namely the *CCS* compendium, in which large synthetic molecules are however barely represented so far.^147,148^

**Figure 7:**
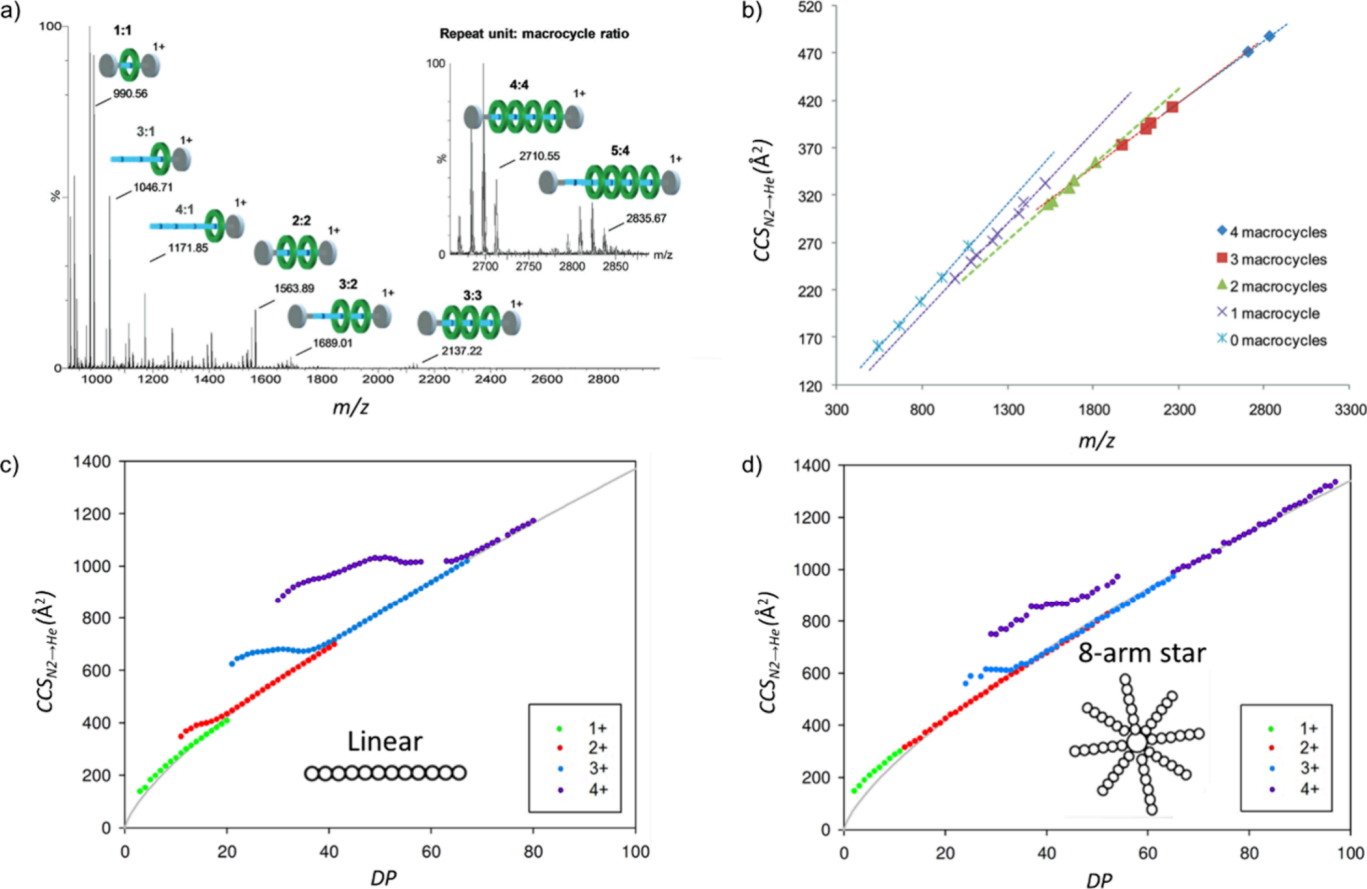
(a) MS spectrum of the polyrotaxane system studied by Scarff et al.;^151^ multiple polyrotaxane and pseudopolyrotaxane species were observed. The polymeric repeat unit:macrocycle ratio is indicated. Inset: Zoom in the 2600–2900 *m*/*z* region. (b) *CCS*_*N2→He*_ values for polyrotaxane species and fragment ions with different numbers of associated macrocycles vs their *m*/*z* ratio. The experimental error is estimated at ca. ± 2%, which is less than the size of the data point markers used within this figure. (c and d) *CCS*_*N2→He*_ values of polymer chains as a function of the degree of polymerization (*DP*) for four charge states of the linear (c) and 8-arm star (d) topoisomers. Reproduced from refs (151), © 2012 American Chemical Society and (152), © 2014 American Chemical Society.

We have used a *CCS/m* plot to assess the structure of synthetic dendrimers^132^ and polymetallic complexes,^57^ showing for the latter that we are able to differentiate closed and open topologies. Von Helden and co-workers were able to distinguish chains, rings and compact structures of POMs, in combination with DFT and gas phase infrared spectroscopy.^149^ For mechanically interlocked polymers, Hanozin et al.^150^ and Scarff et al.^151^ applied a similar workflow. The latter example involves rotaxane polymers consisting of a polymeric backbone of repeating dialkenyl ammonium units, a number of crown ether macrocycles and bulky stopper groups. ^1^H NMR spectroscopy confirmed the rotaxane formation on average; however, the stoichiometries and topologies of the different species could naturally not be explored in detail. The authors used both ESI and MALDI to ionize the analyte mixture, and a range of polymers with 1:1 to 5:4 ratios of repeating unit:macrocycle were found (Figure 7a). IM was applied to assess the ions’ conformational landscape, and the relationship between *CCS*_*N2→He*_ and mass yielded different trends, depending on the number of macrocycles present (Figure 7b). For a constant number of macrocycles, the *CCS*_*N2→He*_ increased linearly with mass, which is expected for the one-dimensional growth of a polymer. Noteworthy is that different lines were found for different numbers of macrocycles, and this is related to the packing density of the ions expressed as the ratio of *CCS*_*N2→He*_ to mass. Polymers with higher numbers of macrocycles yielded lower *CCS*_*N2→He*_ values than those with less macrocycles of the same mass, suggesting that the packing density of the threaded rotaxane units is higher compared to those that are unthreaded. This simple relationship can be used to determine the number of macrocycles for polymers of this family.

The *CCS/m* correlation also was exploited by Morsa et al., probing the topology of one linear and three star-branched poly-ε-caprolactones (PCL).^152^ When plotting the *CCS*_*N2→He*_ values of their different charge states vs the degree of polymerization (*DP*), the authors found significant differences for the *CCS*_*N2→He*_ value depending on whether *DP* is high or low. For high *DPs*, all different charge states collapse onto a common curve similar for each topoisomer, which was fitted with the power equation *CCS*_*N2→He*_*= A · (DP)*^*B*^ and yielded *B* = 0.71 ± 0.01. The authors concluded that this is in good agreement with *2/3*, which for a totally spherical shape is the expected value of *B*.^145^ This supports the idea that long polymer chains adopt approximately spherical conformations, independently of the polymer topology. For the species with lower *DP*, particularly the higher charge states show *CCS*_*N2→He*_ values above the aforementioned curve, suggesting less dense structures. Here, significant differences were found between the topoisomers, as shown in Figure 7c, d for the linear and 8-arm star topoisomers, respectively. The *CCS*_*N2→He*_ expansion describes the difference of the obtained *CCS*_*N2→He*_ value to those predicted from the power equation curve, and for the species with charge states +2 to +4. The general trend suggests smaller *CCS*_*N2→He*_ expansions for the higher branched structures, attributable to lower flexibility in the chain, and offers diagnostic fingerprints to distinguish the topoisomers.

### 3.7 Toward an Experimental IM-MS Framework to Discern Synthetic Architectures

**Figure 8:**
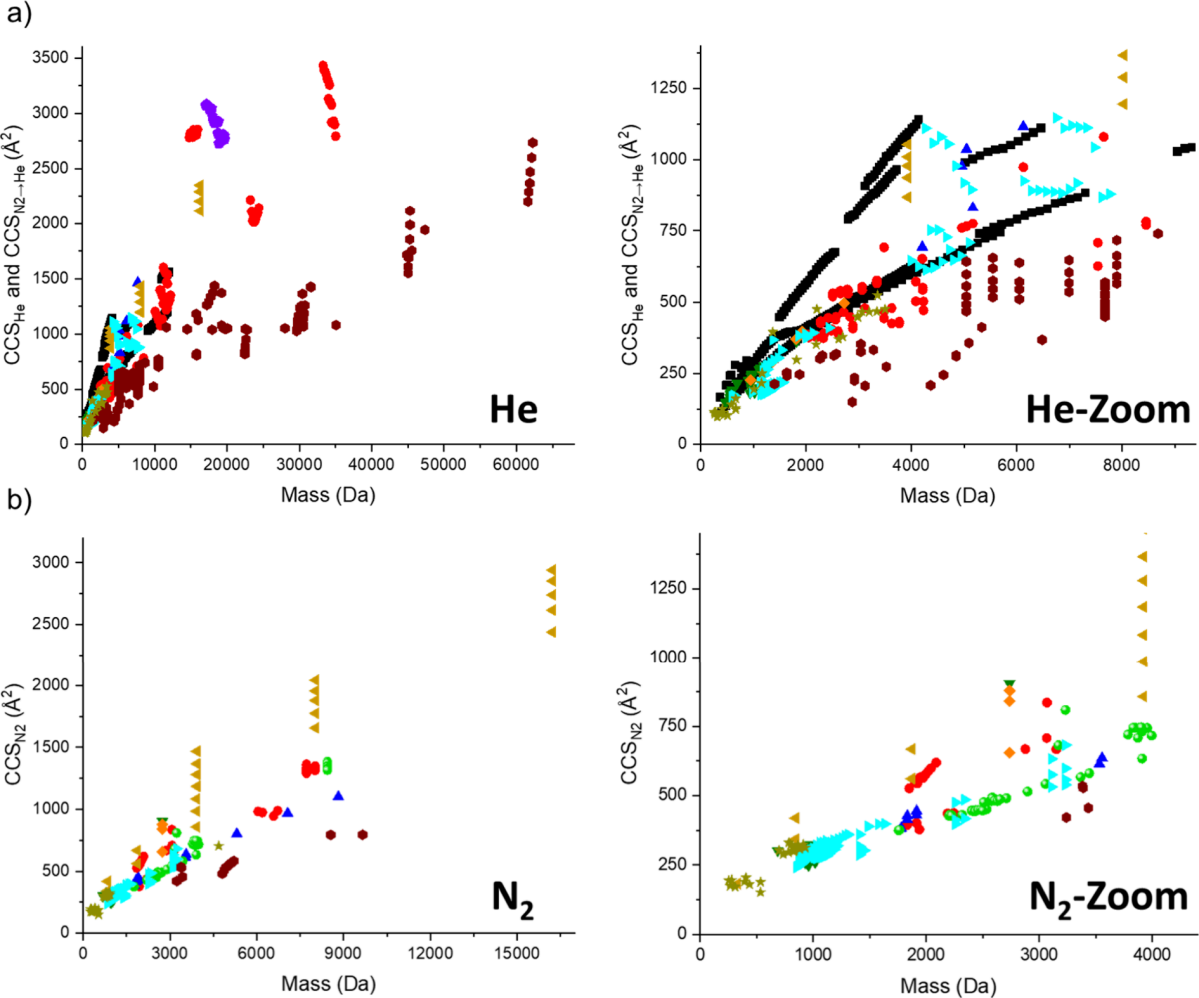
Relationship of *CCS* vs *m* (packing density plot) for (a) *CCS*_*He*_ and *CCS*_*N2→He*_ and (b) *CCS*_*N2*_ values of synthetic molecules (left: full graph, right: zoom), including polymetallic cages (2D and 3D, red circle), polymetallic 2D architectures (purple pentagon), polymetallic host–guest complexes and rotaxanes (light green bowl), polymetallic open structures (blue upward triangle), metallo-nanoclusters (wine hexagons), macrocycles (dark green downward triangle), host–guest complexes and rotaxanes (cyan rightwards triangle), 2D architectures (beige leftwards triangle), interlocked molecules (orange standing square), polymers (black square) and miscellaneous species (dark yellow stars). Experimental *CCS* values throughout this manuscript are denoted with the buffer gas they were measured in, such as *CCS*_*N2*_, *CCS*_*He*_ or *CCS*_*N2→He*_, according to the agreed nomenclature.^11^ Experimental IM method and other parameters can be found in the interactive plot and the corresponding data table under https://chart-studio.plotly.com/∼f33579ng/5/#/ (Helium) and https://chart-studio.plotly.com/∼f33579ng/6/#/ (Nitrogen).

The packing density plots shown above can not only distinguish similar ions from each other and assign them to different families but also provide insights to architectural differences of various compound types. We have constructed a database of 1030 synthetic ions (He: 721, N_2_: 309) from 49 different studies, and plotted *CCS* values vs mass to explore how the topology and molecular architecture determines the *CCS/m* slope and hence the packing density (Figure 8).

For the two common IM gases (helium: Figure 8a and nitrogen: Figure 8b), we found that metallo-nanoclusters (wine hexagons) show the smallest *CCS/m* slope and hence highest packing density. On the other end of the density scale are self-assembled, two-dimensional architectures (beige leftwards triangles and purple pentagons), such as dendrimers or polymetallic snowflakes, giving a particularly high *CCS/m* slope. The data also confirm that filled polymetallic structures (host–guest complexes and rotaxanes, light green bowls) are more dense than hollow polymetallic cages (red circles) and polymetallic open structures (blue upward triangles); however this does depend on the specific ions. No significant differences were observed between polymetallic species and those without heavy metals (e.g., for host–guest complexes and rotaxanes), suggesting that their density and hence their gas phase structures are similar, although this again depends on the specific architecture. Care should be taken when considering structures with d- and f-metals in these plots, as heavy atoms contribute disproportionally to the mass of the ion without affecting the volume in the same magnitude. This suggests that polymetallic species may actually be less dense than shown in Figure 8.

Further density trends are hard to describe, as the structures are highly diverse and difficult to categorize, and a good illustration of this circumstance are the three different polymer families plotted in Figure 8a (black squares), which already cover a wide range of packing densities. We were also interested in how the density relates to those of biomolecules such as native proteins studied in our previous works,^146,153^ and found proteins to have a relatively low *CCS/m* slope and high density, although they are less dense than the remarkably packed metallo-nanoclusters. Another interesting attribute is that the *CCS* values of two-dimensional architectures and nanoclusters show a strong charge-state dependency (multiple data points at highly similar mass, but different *CCS*), which is most likely driven by Coulombic repulsion and expansion as a response to high charge states. Taken together, we provided an experimental framework to understand the packing density of synthetic molecules, and by comparison to the data in Figure 8, we can now evaluate the packing density of a given ion, and potentially attribute it to a single compound family or a group of related architectures. One idea to exploit this further would be to correlate *CCS* values, both experimental and theoretical, with the confined space (geometric) within hollow synthetic architectures. Understanding this relationship would enable the prediction of cavity size based on IM-MS measurements, which would be beneficial for supramolecular and pharmaceutical chemists.

## 4 Disassembly of Molecular Architectures via Tandem Mass Spectrometry

### 4.1 Quantification of Ion Stabilities

The design of synthetic molecules can be challenging, and understanding the fundamental interactions between different building blocks is highly important. This relates to the overall stability of a complex, which defines how easy it is to form a given compound and in what yield, and hence how feasible its use is for future synthetic approaches. Quantifying the stability of compounds with bulk phase methods, e.g. via thermodynamic/kinetic dissociation or exchange experiments, is not trivial and MS^2^ offers a unique approach to this challenge. Working with low analysis times and amounts of sample, MS^2^ evaluates the ion’s stability *in vacuo*, which can be helpful in deciphering the complex properties from those occurring in combination with solvent and counterion effects. The activation in MS^2^ is most commonly realized via collisions with an inert gas (CID), as discussed above, and depending on the collision energy and the ion’s stability, this is often followed by fragmentation.^8,10^ Guided ion beam mass spectrometers, pioneered by Armentrout and co-workers, carefully control the kinetic energy of the collision event. Such experiments produce data that can be extrapolated to a single collision, which makes the derivation of purely thermodynamic data possible.^154−156^

**Figure 9:**
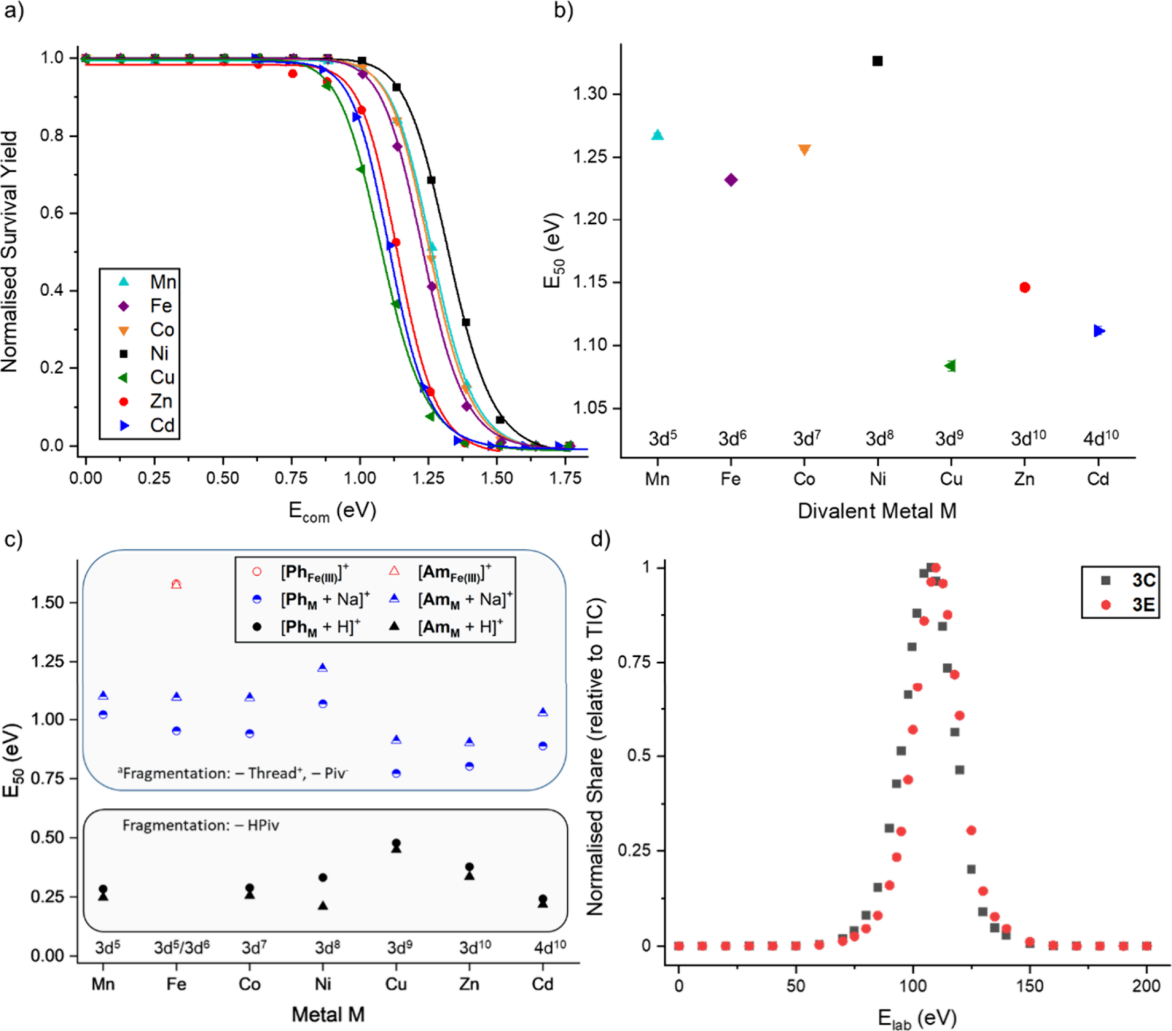
(a) Normalized survival yield vs *E*_com_ for [**Ring**_**M**_]^−^ fitted to a sigmoidal Hill function (M = Mn: cyan, Fe: purple, Co: orange, Ni: black, Cu: green, Zn: red, Cd: blue). (b) *E*_50_ values for [**Ring**_**M**_]^−^ with respect to M. (c) *E*_50_ values for [**Ph**_**M**_ + A]^+^ and [**Am**_**M**_ + A]^+^ (A^+^ = H^+^, Na^+^) with respect to M, as well as the *E*_50_ values of [**Ph**_**Fe(III)**_]^+^ and [**Am**_**Fe(III)**_]^+^. ^a^For the ion [**Am**_**Cu**_ + Na]^+^, the main fragmentation channel is – Cu^II^, – 2 Piv^–^. (d) Share of **3C** (black) and **3E** (red) in dependence of *E*_*lab*_ applied to the precursor [**Ring**_**Mn**_]^−^. In this analysis, we compare the share of the *m*/*z*- and IM selected peaks relative to the total ion count at each *E*_*lab*_. Reproduced from ref (56), © 2022 The Authors.

In most mass spectrometers, performing CID on any given ion involves multiple collisions, which means that both kinetic and thermodynamic effects interfere. Nevertheless, the common CID workflow offers reliable quantification of energetics between similar compounds and can hence be applied to quantify stability. This is most commonly realized via the *E*_50_ value of an ion, which describes the collision energy needed to fragment 50% of the precursor ion. This value is obtained by fitting the curve of the survival yield (how much of the precursor ion “survives” the collisions; per definition between 0 and 1) vs the applied collision energy in the center-of-mass frame (Figure 9a).^157−159^

For example, we have previously used the *E*_50_ values to probe the stability of the polymetallic rings and rotaxanes discussed above.^56^ Our results suggested that the stability of these architectures depends on the d-metal composition and their oxidation states as well as the stopper groups of the threads in the rotaxanes. A very instructive example of the influence of the d-metal composition is the heterometallic ring anions [**Ring**_**M**_]^−^, where M = Mn^II^, Fe^II^, Co^II^, Ni^II^, Cu^II^, Zn^II^, Cd^II^ as described above. We determined their *E*_50_ values, showing that [**Ring**_**Ni**_]^−^ is the most stable species, in fact 22% more stable than the most fragile species [**Ring**_**Cu**_]^−^. This is interesting from a practical perspective for synthetic applications, but also offers a range of fundamental information on the nature of their building blocks. We rationalized the obtained trends (Figure 9b) with arguments from crystal field theory in an octahedral ligand field, such as Jahn–Teller effects.^56^ We extended this study to heterometallic rotaxanes [NH_2_RR′][**Ring**_**M**_], with R = R′ = (CH_2_)_6_NHC(O)^*t*^Bu for **Am**_**M**_ and R = CH_2_C_6_H_5_, R′ = (CH_2_)_2_C_6_H_5_ for **Ph**_**M**_. When considering the sodiated adducts of these neutral species, they followed similar trends, although small deviations were observed (Figure 9c). The protonated species gave a completely different d-metal trend, where **Am**_**Cu**_ and **Ph**_**Cu**_ were found to be most stable. This suggests differences in the electron density depending on the charge carrier A^+^ = H^+^, Na^+^, and for the protonated species this follows the Irving–Williams series for the kinetic stability of d-metal aqua complexes. We also found an increased stability of Fe^III^ over Fe^II^, due to more favorable charge–charge interactions with the anionic ligands.

As the fragmentation pathway of the sodiated rotaxanes involves the loss of the thread, we expected that changing the R and R′ groups also alters the stability of the rotaxane complex. We considered rotaxanes with the two threads mentioned above, **Ph**_**M**_ and **Am**_**M**_, which yielded a higher stability for **Am**_**M**_ due to more favorable thread-ring interactions of the amide groups, as well as their higher steric demand.^56^ We also measured the *E*_50_ values of the pseudorotaxanes [NH_2_R_2_][Cr_7_CoF_8_Piv′_16_], (Piv′ = (OOC)CH_2_^*t*^Bu; R = CH_3_(CH_2_)_*n*_, *n* = 0–5, 7), showing that, although all of these species are not particularly stable, a local maximum was reached for *n* = 1 and a minimum for *n* = 3. These data, later complemented by more time-consuming ^1^H NMR experiments, suggested that α- and β-hydrogen interactions (strongest for *n* = 1, as there are three β-hydrogens in an ethyl group) have the highest impact on the stability of the pseudorotaxanes.^160^

### 4.2 Disassembly Pathways—a Window to Molecular Self-Assembly

Besides the quantitative derivation of stability values, the nature of fragments can also inform on fundamental analyte properties. Common MS^2^ workflows only monitor the *m*/*z* of the product ions; however when combined with IM, their structure(s) can be probed in the same experiment.^161^ As outlined above, we used IM-MS to analyze the disassembly process of [**Ring**_**M**_]^−^, and we observed the loss of metal centers along with anionic ligands (Figure 2b). This involved three main fragmentation pathways: for **1**: – M^II^, – 2 Piv^–^; for **2**: – Cr^III^, – 2 Piv^–^, – F^–^ and for **3**: – Cr^III^, – 3 Piv^–^. Each of these fragment types showed two different types of *CCS*_*N2*_ distributions: one at lower *CCS*_*N2*_, presenting with a narrow peak shape (compact = C), and one at higher *CCS*_*N2*_, with a much broader distribution (extended = E). The population of C and E depend on three factors: the collision energy, the fragment type **1**, **2** or **3**, and the divalent metal M^II^ present in the precursor [**Ring**_**M**_]^−^. Most curiously, we found that for fragment **1** = [Cr_7_F_8_Piv_14_]^−^, there are differences in the population between **C** and **E** when varying the metal M, although M is not even present anymore. This points toward a strong dependency on the reactant ion, the transition state and most likely the [MPiv_2_] leaving group, which all interplay to determine the dissociation pathway.^56^

Stability can also be quantified for different conformations with the same mass, when coupled to IM.^56,162^ For example, Wesdemiotis and co-workers showed a higher stability for a hexacadmium macrocycle than for its linear isomer using MS^2^ coupled with IM-MS.^161^ Kalenius et al. used collisional in-source activation to track the isomerization reaction of the well-studied Au_25_(SR)_18_ cluster to an isomer found in the gas phase.^163^ We applied this to the formation, rather than dissociation, of the distributions **3C** and **3E**, and plotted their absolute share against the total ion count.^56^ This showed that **3C** forms at lower collision energies than **3E**, likely as less energy is required for this channel that does not involve major perturbation (Figure 9d).

**Figure 10:**
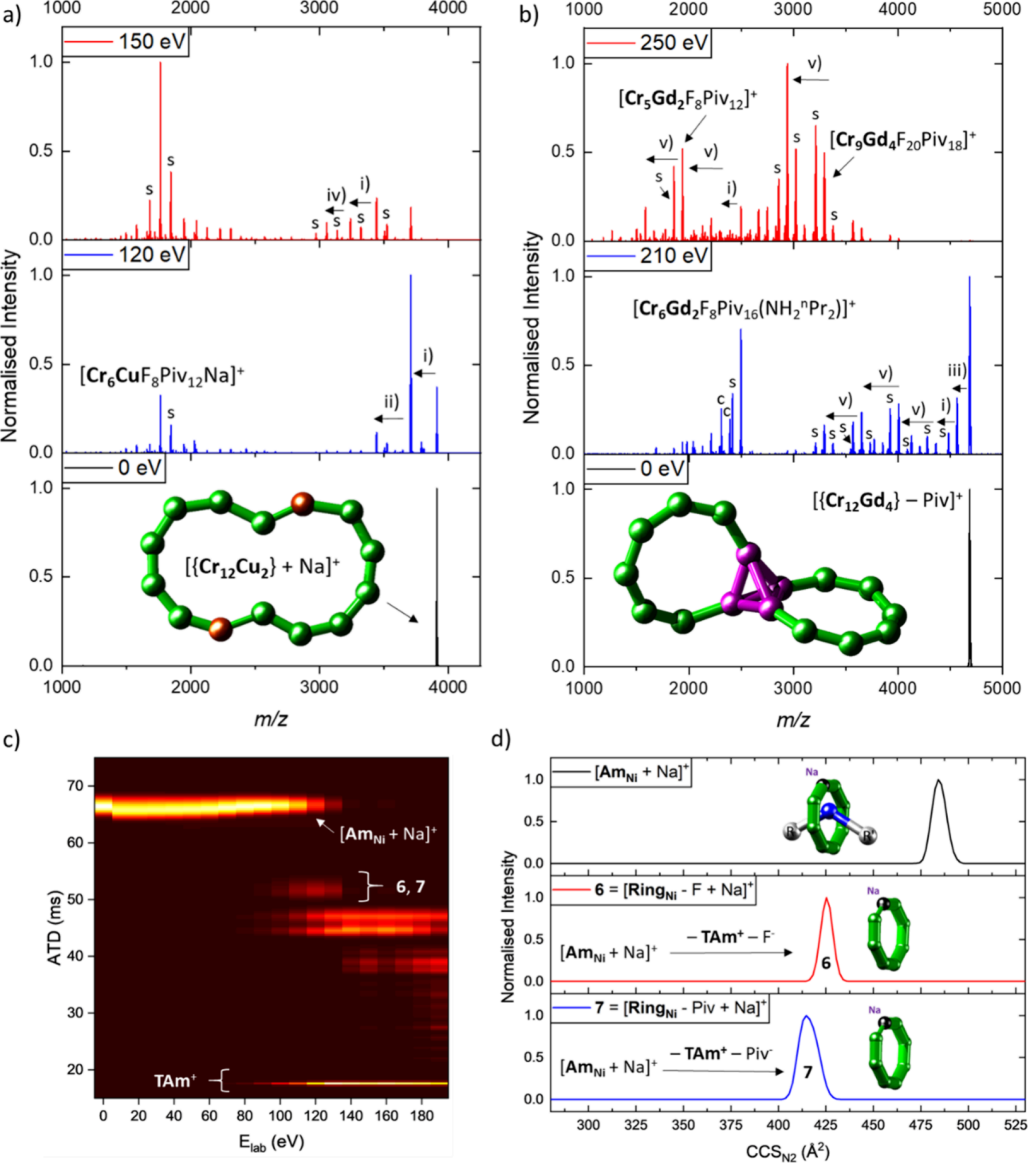
MS^2^ spectra of (a) [{**Cr**_**12**_**Cu**_**2**_} + Na]^+^ and b) [{**Cr**_**12**_**Gd**_**4**_} - Piv]^+^ at different collision energies. Occurring fragmentation pathways are labeled: (i) – [NH_2_^n/i^Pr_2_]^+^, – Piv^–^; (ii) – **Cu**^**II**^, – 2 Piv^–^; (iv) – **Cu**^**II**^, – Piv^–^, – F^–^ and (v) – **Cr**^**III**^, – 3 Piv^–^. Satellite peaks (labeled with “s”) correspond to the labeled main peak via the exchange of one anionic ligand for another one (Piv^–^ for F^–^ or F^–^ for Piv^–^; mass difference = 82 Da). Insets: Schematics of {**Cr**_**12**_**Cu**_**2**_} and {**Cr**_**12**_**Gd**_**4**_} (Cr: green, Cu: brown, Gd: purple). c) Collision energy vs arrival time distribution heat map for [**Am**_**Ni**_ + Na]^+^ as well as the corresponding fragments **6** and **7**. (d) *CCS*_*N2*_ distributions of [**Am**_**Ni**_ + Na]^+^ and its fragments **6**, **7**. Reproduced from ref (56), © 2022 The Authors, and ref (57), © 2023 Springer Nature, with permission under CC-BY 4.0 licenses.

For the disassembly of the larger hourglass ions {Cr_*x*_Cu_2_} (*x* = 10, 12) and the heterocluster {Cr_12_Gd_4_}, which are similarly bridged via F^–^ and Piv^–^ ligands, almost all fragments are present with a closed topology.^57^ All three species disassemble to fragment classes of either low or high mass, leading to a bimodal product ion spectrum (Figure 10a, b). This is produced via stepwise disruption, where one metal center dissociates along with ligands in each step, predominantly at the weak spots/reactive metal sites, or with major disruption and the formation of smaller closed topologies such as {Cr_5_Cu_2_}, {Cr_6_Cu} or {Cr_6_Gd_2_}. Why these species form and why they are preferred over others is beyond the scope of this perspective and can be found in the primary text;^57^ however all results taken together suggest that the disassembly of this family of polymetallic complexes involves two competing disassembly routes.

A critical question is—why is the disassembly mechanism important to understand, and what can we learn from how molecules break? The disassembly mechanisms can teach us about underlying assembly mechanisms, although both do not necessarily correlate and might vary in different phases and under different conditions. Assembly is nevertheless crucial for the design of molecular architectures, and a fundamental scientific assembly problem with relevance to bulk phase chemistry is the question whether a given threaded molecule is a rotaxane, where the thread cannot slip off and thread loss must be accompanied by ring opening, or a pseudorotaxane, where the stopper groups are not large enough to hold the ring on the thread. This topic has been discussed controversially for decades, and IM-MS and MS^2^ can contribute to that question.^164−166^ For the polymetallic rotaxanes **Ph**_**M**_ and **Am**_**M**_, we tracked the arrival time distribution of the *m*/*z*-selected rotaxane ion in response to collision energy, as shown in Figure 10c for [**Am**_**Ni**_ + Na]^+^. For biomacromolecules, this experiment often indicates major structural change, and particularly for proteins the term “collision-induced unfolding” was coined to describe this behavior similar to denaturation.^167^ The rotaxane ion showed a minor contraction followed by a minor extension at low energies; however, no major changes were observed over the studied range of collision energies. Compelling evidence came from examining the conformational landscape of the product ions **6** and **7**, which are {Cr_7_M} species after the thread is lost. These appear with a narrow peak shape, and comparing their *CCS*_*N2*_ to values known from the octametallic [**Ring**_**M**_]^−^ anions showed a high agreement (Figure 10d). Hence, the fragments after thread loss are closed rings, which suggests that a slipping mechanism occurred for the disassembly of the rotaxanes **Ph**_**M**_ and **Am**_**M**_. This finding was remarkable, as space-filling models of the rotaxanes’ crystal structures indicate that this is not possible, which might suggest that the ring opens upon collisional activation and compacts again on the millisecond time scale. As we have observed the reclosing of rings upon losses of metal centers before, this is a plausible dethreading mechanism.

**Figure 11:**
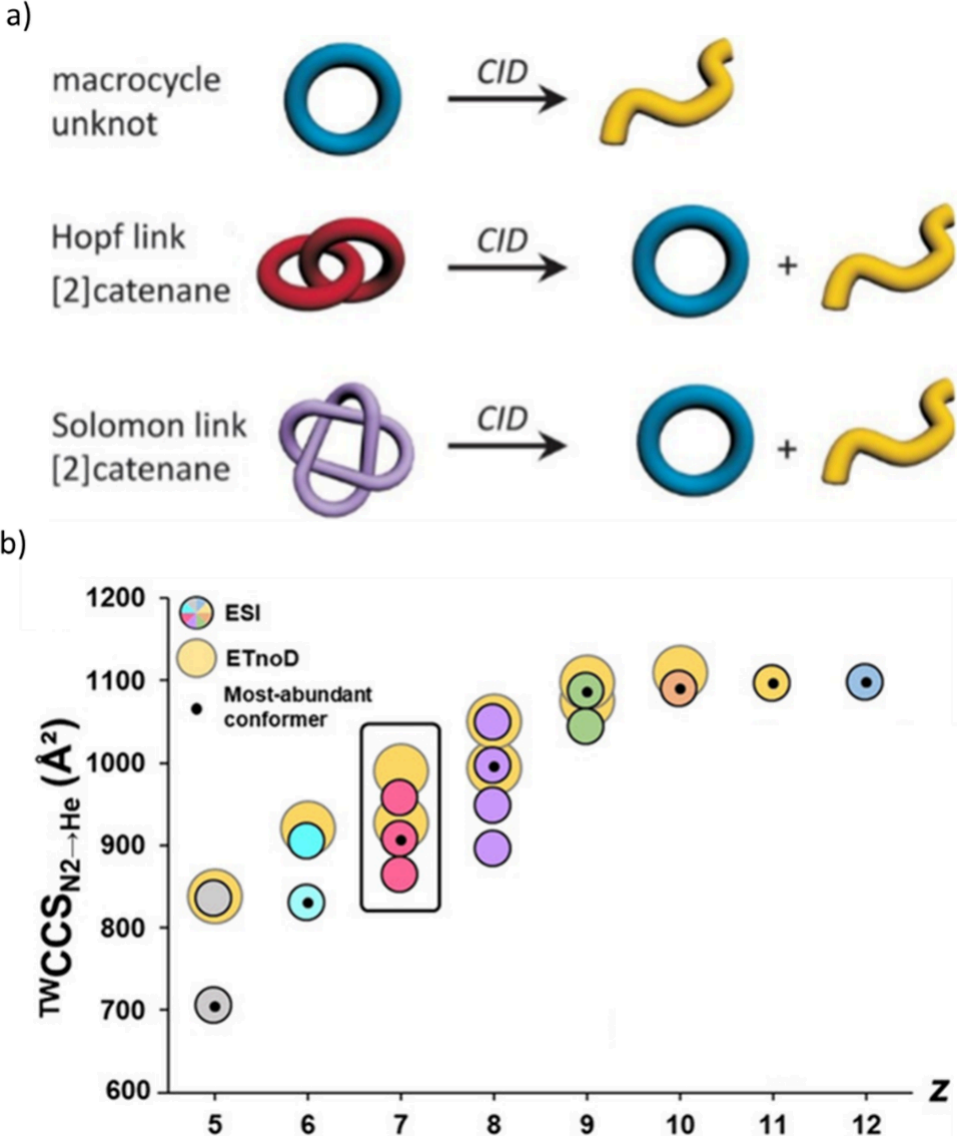
(a) Topologies of the three discussed structures (macrocycle, Hopf link and Solomon link) including the expected fragment topologies after CID experiments. (b) Variation of *CCS*_*N2→He*_ of the studied molecular switch system^169^ with *z* for ESI-generated closed shell species and ETnoD-generated radical species starting from the *z = 11* precursor. Reproduced with permission from ref (168), © 2019 Wiley-VCH Verlag GmbH & Co. KGaA, and ref (169), © 2021 The Authors.

Kruve et al. distinguished the topology of interlocked and knotted molecules by analyzing their disassembly with MS^2^ and IM-MS.^168^ The simplest example studied was the differentiation between a macrocycle and a [2]catenane (consisting of two interlocked macrocycles), and this can be achieved only by MS^2^. In the case of the macrocycle, collisional activation yields a single, (linear) fragment, whereas the fragmentation of the [2]catenane produces two fragments, one ring and one linear (Figure 11a). These products commonly have different masses, and hence the number of peaks in the MS^2^ spectra is often sufficient to distinguish between the two precursor topologies. If the fragments have the same mass (“isobaric”), the unambiguous identification of the precursor topology can be achieved via the number of fragment IM peaks (macrocycle: 1, catenane: 2).

More difficult is the differentiation between two [2]catenanes with different numbers of knots, such as a Hopf link and a Solomon link. Both species yield the same fragment types (one ring and one linear), and the absolute *CCS* values are difficult to rationalize without benchmarking similar systems. This problem was resolved by assessing the relative arrival time difference between the least entangled fragment and the parent ion, and this so-called “floppiness factor (*Fl*)” can be used to inform on precursor topology. The higher the *Fl* is, the more similar are precursor and fragment ions, and hence the less densely packed is the precursor. Kruve et al. found a significantly smaller *Fl* for the Solomon link compared to the Hopf link, confirming that the latter is less densely packed than the former. The authors also extended their work further to more complex systems such as [3]catenanes and trefoil knots, highlighting that their approach is applicable to interlocked and knotted molecules in general.

### 4.3 Combining IM-MS with Other Tandem Mass Spectrometry Techniques

While the vast majority of MS^2^ experiments on synthetic molecules employed CID, other activation methods such as light (UV, IR) or electrons have their merit and are well explored for biological systems.^8,10^ In a recent work, Hanozin et al. investigated the impact of electron transfers on the conformational landscape of a molecular switch.^169^ Electron-transfer dissociation (ETD) is a commonly used MS^2^ technique, in which electrons are transferred from an ionized reagent to the analyte ion. While this often leads to fragmentation, some species tend not to dissociate but rather reduce due to the attachment of electrons (ETnoD), and this allows the investigation of analyte ions in different oxidation and charge states. To monitor the structure of the molecular switch, the authors coupled ETnoD with IM (and gas phase IR spectroscopy), showing that the manipulation of the charge state leads to coconformational transitions as part of a switching motion, which are similar, but slightly distinct to charge state differences in usual MS (Figure 11b). As the influence of external stimuli, such as electrons, is a central question when it comes to molecular switches, ETnoD coupled with IM-MS has notable merit in assessing this motion. Taken together, we conclude that MS^2^ is a suitable tool to probe the stability and conformational transitions of synthetic molecules, and particularly when coupled to IM-MS it can inform on their properties via the investigation of their disassembly pathways.

## 5 Conclusions

We have demonstrated how IM-MS is employed to characterize the structure of synthetic complexes, form and predict novel complexes in the gas phase, and investigate the stability and disassembly pathways of molecular architectures. IM-MS offers the chemist a laboratory where they can study isolated analytes, with precise control over the number of counterions and ligands. For a given compound class, this can permit dissection of the effect of any subunit on intrinsic properties such as structure and stability. For synthetic chemists, a primary motivation to use IM-MS is structural characterization; however it also readily provides additional information for bulk-phase chemistry regarding the formation and disassembly of complexes (Figure 1). We have highlighted how the integration of other structural characterization methods with the IM-MS apparatus can provide further insights into the molecules of interest, as well as supporting IM-MS data with those from other methods, including computational modeling. So far the main challenge is the correlation of *CCS* data with atomically resolved structures; however given the robust nature of the *m*/*z* and *CCS* data produced, the ability to compare between laboratories and to generate large data sets with relative ease, we anticipate that machine learning approaches will significantly facilitate structural assignments.

Our experimental framework uses *m*/*z* and *CCS* data to assess packing density and in turn to distinguish the topological preferences of different classes of synthetic molecules (Figure 8). We predict that the full potential of IM-MS for synthetic supramolecular chemistry will be best realized in its use for large complexes, where reliable structures from computational modeling, X-ray crystallography and NMR spectroscopy are difficult to obtain.^2^ Extremely large complexes can be transferred to the gas phase and analyzed with MS, as evidenced by data from mega- and even gigadalton ions of viral capsids.^170^ Larger (synthetic) complexes can be harder to preserve than biomacromolecules of equivalent *m*/*z*, which suggests more development of ion sources and transfer optics may be required. While first steps have been taken to establish IM-MS for larger architectures, including a {Cd_24_} cubic star by Chan and co-workers (≈ 35 kDa)^77^ and a {Te_8_W_116_}_2_ polyoxometalate by Surman, Rubbins et al. (≈ 62 kDa, to the best of our knowledge the largest synthetic molecule analyzed with IM-MS to this day),^126^ the majority of IM-MS work of synthetic molecules has been done on small building blocks. We conclude that, driven by constantly improving instrumentation and method development, the characterization of synthetic molecules using IM-MS will attract more attention, particularly for larger complexes.

## Data Availability

The supporting data referred to in Figure 8 of this manuscript is contained within interactive plotly Chart Studio plots available under https://chart-studio.plotly.com/∼f33579ng/5/#/ (Helium) and https://chart-studio.plotly.com/∼f33579ng/6/#/ (Nitrogen).
